# RankProd 2.0: a refactored bioconductor package for detecting differentially expressed features in molecular profiling datasets

**DOI:** 10.1093/bioinformatics/btx292

**Published:** 2017-05-08

**Authors:** Francesco Del Carratore, Andris Jankevics, Rob Eisinga, Tom Heskes, Fangxin Hong, Rainer Breitling

**Affiliations:** 1 Faculty of Science and Engineering, Manchester Institute of Biotechnology, University of Manchester, Manchester, UK; 2 Department of Electrical Engineering and Electronics, University of Liverpool, Liverpool, UK; 3 Department of Social Science Research Methods, Radboud University Nijmegen, Nijmegen, GD, Netherlands; 4 Institute for Computing and Information Sciences, Radboud University Nijmegen, Nijmegen, EC, Netherlands; 5 Harvard School of Public Health, Dana-Farber Cancer Institute, Boston, MA, USA

## Abstract

**Motivation:**

The Rank Product (RP) is a statistical technique widely used to detect differentially expressed features in molecular profiling experiments such as transcriptomics, metabolomics and proteomics studies. An implementation of the RP and the closely related Rank Sum (RS) statistics has been available in the RankProd Bioconductor package for several years. However, several recent advances in the understanding of the statistical foundations of the method have made a complete refactoring of the existing package desirable.

**Results:**

We implemented a completely refactored version of the RankProd package, which provides a more principled implementation of the statistics for unpaired datasets. Moreover, the permutation-based *P*-value estimation methods have been replaced by exact methods, providing faster and more accurate results.

**Availability and implementation:**

RankProd 2.0 is available at Bioconductor (https://www.bioconductor.org/packages/devel/bioc/html/RankProd.html) and as part of the mzMatch pipeline (http://www.mzmatch.sourceforge.net).

**Supplementary information:**

Supplementary data are available at *Bioinformatics* online.

## 1 Introduction

Finding differentially expressed molecular features when comparing different conditions plays a pivotal role in all kinds of molecular profiling studies (‘omics’). The Rank Product (RP) and the Rank Sum (RS) are two non-parametric statistics widely used to detect variables consistently upregulated (or downregulated) in replicate experiments (Breitling and Herzyk, 2005; Breitling *et al.*, 2004). Originally developed for the analysis of gene expression microarrays, both methods are more accurate and powerful than their usual competitors in a number of different scenarios (e.g. abnormally distributed noise, heterogeneity of samples, small fraction of changed features, small sample size), as demonstrated in extensive numerical studies (Breitling and Herzyk, 2005; Jeffery *et al.*, 2006; Koziol, 2010a, b). The main identified weakness of the RP method is its sensitivity to variable-specific measurement variance. Nevertheless, this problem has been successfully addressed by a number of variance stabilizing normalization techniques (Breitling and Herzyk, 2005; Durbin *et al.*, 2002; Huber *et al.*, 2002). An R Bioconductor package implementing RP and the closely related RS has been available and widely used for several years (Hong *et al.*, 2006). However, recent improvements in our understanding of the two statistics made a refactored version of the package desirable. In the old implementation, the *P*-value estimation had been performed by a permutation-based method for both statistics (Hong *et al.*, 2006). This method requires a computationally demanding number of permutations in order to obtain accurate results and, when dealing with the tails of the distribution (i.e. the most interesting molecular features), the estimates are particularly unreliable. In RankProd 2.0, this limitation has been successfully tackled. Regarding the RP, the *P*-value estimation is now performed by applying the fast method proposed by Heskes *et al.* (2014). This tailor-made solution calculates strict bounds and very accurate approximate *P*-values for RP analysis. For the RS, a new exact method for the evaluation of the *P*-values has been developed and implemented as described in **Section 3**. The RP was initially introduced for the analysis of gene expression in paired datasets, specifically two-color microarrays (Breitling *et al.*, 2004). Nevertheless, the old RankProd package provided an *ad hoc* strategy to cope with unpaired datasets. Provided that unpaired datasets are increasingly common, we developed a more principled approach described in **Section 4**, which provides a more reliable application of RP and RS in the analysis of unpaired datasets.

## 2 *P*-values estimation for the RP

The *P*-value estimation for the RP has been intensely studied in the last few years. Koziol (2010a, 2016) approximated the distribution of the RP with a gamma distribution. Such approximation resulted to be imprecise when dealing with the tails of the distribution (Eisinga *et al.*, 2013). Eisinga *et al.* (2013) derived the exact probability distribution of the RP statistic. Unfortunately, this is time-demanding and impractical to use with large datasets. For this reason, we chose the method proposed by Heskes *et al.* (2014), which allows a very accurate approximation of the *P*-values in a computationally fast manner. This method allows us to calculate strict bounds for the exact *P*-values and extremely accurate estimates by considering the geometric mean of the upper and lower bounds. This approach significantly speeds up the RP analysis. When considering a typical paired dataset (*N* = 1000 and *K* = 10), the computation time is now reduced by a factor of ∼500, when compared with the analysis performed with the previous approach (using 10 000 permutations).

## 3 *P*-values estimation for the RS

Previously, the only method available to estimate the *P*-values for the RS statistic was the permutation-based approach already implemented in the RankProd package (Hong *et al.*, 2006). Here we introduce a method for the exact calculation of the RS *P*-values. This is derived from the simple observation that under the null hypothesis, the probability distribution of the RS, in an experiment with *N* variables and *K* replicates, is exactly the same as the probability distribution of the sum of the outcomes obtained by rolling *K* dice with *N* faces (http://mathworld.wolfram.com/Dice.html). The implementation of this approach notably speeds up the RS analysis. When considering a typical paired dataset (*N* = 1000 and *K* = 10), the computation time is now reduced by a factor of ∼1200, when compared with the analysis performed with the previous approach (using 10 000 permutations). When the size of the dataset is such that the time needed to evaluate the exact *P*-values becomes unacceptable, the new package uses the exact distribution for the tails of the distribution only, whereas all the other *P*-values are evaluated through a very accurate Gaussian approximation. The extent of the tails and the threshold used to switch between the two strategies are determined by the heuristic rule described, together with the details of the calculation, in the Supplementary Material.

## 4 Application to unpaired datasets

The previous version of the RankProd package provided an *ad hoc* approach to analyze unpaired datasets. This approach consists in considering all the possible pairs that can be obtained from the unpaired samples. Conversely, our new approach computes a user-defined number of random paired datasets and evaluates the RP (or RS) statistic per each of them. Each of these randomly paired datasets has the same size as if the experiment had originally been performed in a paired design. For each variable, the final RP (or RS) value returned is the median of all the values found during the random pairing process. The *P*-values are then computed as in the case of a paired experiment. A detailed description of this new approach can be found in the Supplementary Material.

## 5 Conclusion

The RankProd 2.0 package provides a robust and reliable implementation of the RP methods. Unpaired datasets are now handled through a new approach that significantly improves the performance of the methods. The *P*-value estimation for the RP is now faster and much more accurate, while for the RS we introduced a new and fast method able to evaluate the exact *P*-values. Full backward compatibility has been kept despite the complete refactoring. This improved implementation allows a more reliable application of these methods across the full spectrum of modern molecular profiling technologies. The new implementation of the method has also been integrated in the mzMatch pipeline (Scheltema *et al.*, 2011).

## Funding

This work was supported by the BBSRC [BB/M017702/1]; ‘Centre for synthetic biology of fine and speciality chemicals’.

*Conflict of Interest*: none declared.

## Supplementary Material

Supplementary Data
