# Supplementary material for: RankProd 2.0: a refactored bioconductor package for detecting differentially expressed features in molecular profiling datasets
Source: Bioinformatics. 2017 May 8;33(17):2774–5. doi: 10.1093/bioinformatics/btx292 (PMC5860065; doi:10.1093/bioinformatics/btx292)
Supplement: Supplementary Data [file btx292_supplementary_rpsupplement.pdf]

Bioinformatics
doi.10.1093/bioinformatics/xxxxxx
Advance Access Publication Date: Day Month Year
Applications note

OXFORD

Gene expression

RankProd 2.0: a refactored Bioconductor package for detecting differentially expressed features in molecular profiling datasets—Supplementary Material

Francesco Del Carratore [1], Andris Jankevics [2], Rob Eisinga [3], Tom Heskes [4], Fangxin Hong [5] and Rainer Breitling [1,*]

[1] Manchester Institute of Biotechnology, Faculty of Science and Engineering, University of Manchester, Manchester, M1 7DN, UK
[2] Department of Electrical Engineering and Electronics, University of Liverpool, Liverpool, L69 3GJ, UK
[3] Department of Social Science Research Methods, Radboud University Nijmegen, Nijmegen, 6525 GD, Netherlands
[4] Institute for Computing and Information Sciences, Radboud University Nijmegen, Nijmegen, 6525 EC, Netherlands
[5] Dana-Farber Cancer Institute, Harvard School of Public Health, Boston, MA 02115, USA

* To whom correspondence should be addressed.
Associate Editor: XXXXXXX

Received on XXXXX; revised on XXXXX; accepted on XXXXX

Abstract

1 Description of the Rank Product and Rank Sum methods

The Rank Product (RP) and the Rank Sum (RS) are two non-parametric statistics used to detect features consistently upregulated (or downregulated) in a number of replicate experiments. Initially proposed by Breitling *et al.* (2004) for the analysis of gene expression microarray data, the Rank Products method is derived from biological reasoning and is based on relatively weak assumptions:

- **Assumption 1:** Among all the measured features, only a few molecular species are differentially expressed;
- **Assumption 2:** The measurements are independent between replicate experiments;
- **Assumption 3:** Most of the changes are independent of each other;
- **Assumption 4:** The variance is about equal for all features.

Suppose we have an omics dataset where a total of N molecular features (e.g. genes, proteins, metabolites) have been measured in K replicated experiments. Let $r_{i,j}$ be the position of the ith feature in the jth replicate experiment in a list ordered according to fold changes (in decreasing order if we are interested in upregulated features, or in increasing order vice versa). Under the null hypothesis (no differentially expressed molecular features are present in the dataset), the position of a feature in the list generated by one replicate comes from a uniform distribution (i.e. $P(r_i = x) = 1/N$, where $x \in \{1, .., N\}$). Considering all the K replicates, one should notice that it is extremely unlikely to find the same feature at the top of each list just by chance. In fact the exact probability of a gene being ranked 1 in each replicate is exactly $1/N^K$. The RP (Rank Product) statistic for the ith feature is defined as the geometric mean of all the ranks of the feature obtained in each replicate:

$$RP_i = \left(\prod_{j=1}^{K} r_{i,j} \right)^{1/K}; \qquad (1)$$

the related RS (Rank Sum) statistic is defined as the arithmetic mean of all the ranks:

$$RS_i = \frac{1}{K} \sum_{j=1}^{K} r_{i,j}. \qquad (2)$$

The molecular features with the smallest RP (or RS) value are the most likely to be upregulated or downregulated (according to the order we chose when ranking the fold changes and under the assumptions enlisted above). Detailed comparative evaluations of the performance of RP and RS on simulated and real experimental datasets have been published by Breitling *et al.* (2004), Breitling and Herzyk (2005a), Breitling and Herzyk (2005b), Jeffery *et al.* (2006), Koziol (2010a) and Koziol (2010b).

© The Author 2016. Published by Oxford University Press. All rights reserved. For permissions, please e-mail: journals.permissions@oup.com

2 P-values evaluation

Selecting the variables with the smallest RP (or RS) values for further study can be sufficient, but it is of great interest knowing how statistically significant the changes are and how many of the selected variables are likely to be truly differentially expressed. In other words, the evaluation of the p-values associated with the RP and RS values plays a key role in the interpretation of the results. In the original version of the method, the determination of significance levels was based on a simple but time-consuming permutation-based estimation procedure both for the RP and the RS (Breitling *et al.*, 2004). This method is based on the generation of a large number of random experiments with the same number of replicates and features (as in the real data) in order to evaluate the percentage of false positives (pfp):

1. generate p permutations of K ranked lists of dimension $(1 \times N)$;
2. calculate the RP (or RS) values of the N features in each permutation;
3. let c be the number of times the rank products of the ith feature in the permutations are smaller or equal to the observed rank product value;
4. evaluate the expected value for the rank product: $E(RP_i) = \frac{c}{p}$;
5. evaluate the percentage of false positives: $\text{pfp}_i = \frac{E(RP_i)}{\text{rank}_i}$, where rank_i is the position of the ith feature in a list sorted by increasing rank products.

Using this procedure automatically allows to cope with the multiple testing problem. Especially when dealing with a large number of features, as is typical in modern molecular profiling experiments, to get a reliable estimate this method requires a computationally demanding number of permutations. To tackle this problem, Koziol (2010a) used a gamma distribution to approximate the distribution of the rank products. This considerably speeds up the calculations, but it has been shown that this approximation is imprecise when dealing with the tails of the distribution, i.e. the most interesting (most strongly changed) features (Eisinga *et al.*, 2013). Eisinga *et al.* (2013) derived the exact probability distribution of the Rank Product statistic. The use of the exact p-values offers a significant improvement both over the gamma approximation and the permutation approximation, especially regarding small p-values in the tails.

Unfortunately, the evaluation of the exact p-values is computationally time demanding, especially with large numbers of features and replicates. For this reason, in the new version of the package the evaluation of the p-values for the RP statistic uses an alternative method proposed by Heskes *et al.* (2014), which allows a very accurate approximation of the p-values in a computationally fast manner by computing strict bounds for the exact p-values and approximating the latter as the geometric mean of the bounds. Regarding the RS, we have incorporated a new method to compute the exact p-value. It is easy to understand that, under the null hypothesis, the probability distribution of the RS, in an experiment with N features and K replicates, is equivalent to the probability distribution of the sum of the outcomes obtained by rolling K dice with N faces. This common challenge of traditional statistics has a long story that goes back to the 16th century. It was solved by enumeration for two and three ordinary dice in Cardano's *Liber de Ludo Aleae* (Cardano, 1663, p: 264-265), written in 1564 and published many years later (Bellhouse, 2005). The same problem was also tackled by Blaise Pascal and Pierre de Fermat in a number of letters that they exchanged in 1654, which mark the beginning of modern statistics (Hald, 2003, p: 67-68). Their findings and additional analyses were published by Huygens (1657, p: 521-534) in *De Ratiociniis in Ludo Aleae*. In the early 18th century, de Moivre (1711) used the generating function to obtain the probability of a sum of s points on a roll of K fair N-sided dice. Concurrently, Rémond de Montmort (1713) provided a combinatorial solution for such distribution. For a detailed historical account see Hald (2003, p: 204-2013). The distribution is described as an exercise or example demonstrating the power of the probability generating function as a problem-solving tool in classic and modern textbooks on statistics (Uspensky, 1937, p: 23-24; Feller, 1968, p: 284-285; Johnson and Kotz, 1977, p: 56-57; Berry and Lindgren, 1996, p: 107-111; DasGupta, 2010, p: 84-85) and combinatorics (Dobrushkin, 2009, p: 396-371), and it is discussed in various statistical papers (Tsao, 1955; Takács, 1967; Murty, 1981; Neff, 1982; Fielder and Alford, 1991; Bollinger, 1986; Balasubramanian *et al.*, 1995; Albert, 2002; Ericksen, 2002; McShane and Ratliff, 2003) and resources (Weisstein, 2016). We describe the exact null in rather detail, as the information drawn together here is available but widely scattered in literature. This may also be one of the reasons the exact distribution has been reinvented many times and in various fields, including genetics (Wise and Lewis, 1999; Levinson *et al.*, 2003), psychology (Edgington, 1972; Strube and Miller, 1986), veterinary science (Reiczigel, 1999), agricultural science (Luo *et al.*, 2010), rank sum testing (Özkan, 1987), gaming research (Singh *et al.*, 2011), and statistics (Shen and Marston, 1995). Wise *et al.* (1999), Albert (2002) and Eger (2013), amongst others, derived the exact null distribution using an inclusion-exclusion argument.

We have K independent rankings (replicate experiments), each with integer-valued ranks $\{1, 2, ..., N\}$ (features). Under the null hypothesis, the random rankings have a discrete uniform distribution with probability $P(R = r) = p_r = N^{-1}$, for $1 \le r \le N$. The generating function of the sequence of probabilities of the random rankings is the finite power series:

$$f(t; N) = p_1 t + p_2 t^2 + ... + p_N t^N = \sum_{r=1}^{N} p_r t^r \tag{3}$$

where the probabilities p_r of the integer rank values r are the coefficients of t^r. This function can be put in compact form using the formula for the sum of integers in a geometric progression (i.e., finite geometric series):

$$f(t; N) = \sum_{r=1}^{N} p_r t^r = \sum_{r=1}^{N} \frac{1}{N} t^r = \frac{1}{N} \sum_{r=1}^{N} t^r = \frac{1}{N} \frac{t(1 - t^N)}{(1 - t)}. \tag{4}$$

From the convolution theorem (e.g., Feller, 1968, p: 267; Johnson *et al.*, 2005, p: 60), the probability generating function of the sum of K mutually independent rankings, each assuming the ranks $\{1, 2, ..., N\}$, is given by the Kth power of $f(t; n)$:

$$f(t; N, K) = \left\{ \frac{1}{N} \frac{t(1 - t^N)}{(1 - t)} \right\}^K = \left(\frac{1}{N}\right)^K t^K \sum_{i=0}^{K} \binom{K}{i} (-t^N)^i \sum_{l=0}^{\infty} \binom{-K}{l} (-t)^l. \tag{5}$$

The first sum on the right is the binomial expansion of $(1 - t^N)^K$ and the second sum is the power series expansion of $(1 - t)^{-K}$. Using the -1 transformation

$$\sum_{i=0}^{K} \binom{K}{i} (-t^N)^i = \sum_{i=0}^{K} (-1)^i \binom{K}{i} (t^N)^i, \tag{6}$$

and the upper negation identity transformation

$$\sum_{l=0}^{\infty} (-1)^l \binom{-K}{l} t^l = \sum_{l=0}^{\infty} \binom{l + K - 1}{K - 1} t^l, \qquad | t | < 1, \tag{7}$$

we obtain:

$$f(t; N, K) = \left(\frac{1}{N}\right)^K t^K \sum_{i=0}^{K} (-1)^i \binom{K}{i} t^{Ni} \sum_{l=0}^{\infty} \binom{l + K - 1}{K - 1} t^l. \tag{8}$$

To calculate the probability $P(S = s; N, K)$ of rank sum s as the coefficient of the power of the auxiliary variable t, we write this product of

sums as a double sum and collect all terms involving powers of t, which gives:

$$f(t;N,K) = \left(\frac{1}{N}\right)^K \sum_{i=0}^{K}\sum_{l=0}^{\infty}(-1)^i \binom{K}{i}\binom{l+K-1}{K-1} t^{K+Ni+l}. \tag{9}$$

With the change of variable $s = k + Ni + l$, the expression becomes:

$$f(t;N,K) = \sum_{s=K}^{NK}\left[\left(\frac{1}{N}\right)^K \sum_{i=0}^{K}(-1)^i \binom{K}{i}\binom{s-Ni-1}{K-1}\right] t^s, \quad \mid t \mid < 1 \wedge t \neq 0 \tag{10}$$

by noting that whenever $l \geq 0$ and $k+Ni+l = s$, we have $l+k = s+Ni$. Only finitely many terms in this sum are different from zero. To obtain these non-zero terms one should note that:

$$\binom{s-Ni-1}{K-1} = \binom{s-Ni-1}{s-Ni-K}. \tag{11}$$

The binomial coefficient on the right is greater than zero only when $s - Ni - K \geq 0$, and this is the case only for $i \leq (s-K)/N$. Hence other terms do not contribute to the summation in $f(t;N,K)$. The probability of obtaining rank sum s is therefore:

$$P(S = s;N,K) = \frac{1}{N^K}\sum_{i=0}^{\lfloor (s-K)/N \rfloor}(-1)^i \binom{K}{i}\binom{s-Ni-1}{K-1}, \quad K \leq s \leq NK \tag{12}$$

where $\lfloor . \rfloor$ is the floor function, i.e., the largest integer less than or equal to $(s-K)/N$.
Its numerator

$$W(s;N,K) = \sum_{i=0}^{\lfloor (s-K)/N \rfloor}(-1)^i \binom{K}{i}\binom{s-Ni-1}{K-1} \tag{13}$$

gives the number of ways to obtain rank sum s, for K mutually independent rankings, each assuming the ranks $\{1, 2, ..., N\}$, i.e., the number of (weak) compositions of an integer $s = r_1 + r_2 + ... + r_K$, where $1 \leq r_i \leq N$, for $i = 1, 2, ..., K$. It may also be described as the number of ways to place s distinguishable balls in K cells so that each cell receives at least 1, but no more than N balls (Albert, 2002; Dobrushkin, 2009, p: 369).
The cumulative distribution of rank sum s has the generating function $f(t;N,K)/(1-t)$ and is obtained as:

$$P(S \leq s;N,K) = \frac{1}{N^K}\sum_{i=0}^{\lfloor (s-K)/N \rfloor}(-1)^i \binom{K}{i}\binom{s-Ni}{K}, \tag{14}$$

by repeated application of Pascal's identity:

$$\binom{m}{r} + \binom{m}{r-1} = \binom{m+1}{r}, \quad 1 \leq r \leq m+1, \tag{15}$$

(Feller, 1968, p: 285; Koziol and Feng, 2004). The probability mass function of the discrete random RSs is symmetric (Tsao, 1955), that is:

$$P(S = K+v;N,K) = P(S = NK-v;N,K), \quad v = 0, 1, ..., (N-1)K. \tag{16}$$

For the probabilities this implies that only half of the values need to be calculated and that the remaining half can be obtained by the identity:

$$P(S = NK-v;N,K) = 1 - P(S = K+v-1;N,K) \tag{17}$$

given in Tsao (1956). In particular, for the number of compositions we have:

$$W(s;N,K) = \sum_{i=0}^{\lfloor (s-K)/N \rfloor}(-1)^i \binom{K}{i}\binom{s-Ni-1}{K-1} = \sum_{i=0}^{\lfloor (NK-s)/N \rfloor}(-1)^i \binom{K}{i}\binom{K(N+1)-s-Ni-1}{K-1} \tag{18}$$

as in Wardlaw (1991), and for the p-values:

$$P(S \leq s;N,K) = \frac{1}{N^K}\sum_{i=0}^{\lfloor (s-K)/N \rfloor}(-1)^i \binom{K}{i}\binom{s-Ni}{K} = 1 - \left\{\frac{1}{N^K}\sum_{i=0}^{\lfloor (NK-s)/N \rfloor}(-1)^i \binom{K}{i}\binom{K(N+1)-s-Ni-1}{K}\right\}. \tag{19}$$

The exact mean and variance of the random RS variable are:

$$E(S) = \frac{1}{2}(N+1)K \qquad \text{Var}(S) = \frac{1}{12}(N^2-1)K. \tag{20}$$

As the distribution is symmetric, the skewness is zero, as are all odd order moments. The excess kurtosis is less than zero (Whitfield, 1953), implying that the distribution has thinner extreme tails than the normal. Various studies have proved, however, that under the null hypothesis the sum of ranks is asymptotically normally distributed (Stuart, 1954; Tsao, 1956; Koziol and Feng, 2004; DasGupta, 2010).
The calculation of the exact p-value of rank sum s given K and N is implemented in R (R Core Team, 2015), as shown in **Algorithm 1**. This

Algorithm 1 Sum (S) of K, N-sided Dice cumulative distribution

procedure dice.cdf(S, N, K)
  $a \leftarrow \text{trunc}((K(N+1)/2))$
  $P \leftarrow 0$
  if $S \leq a$ **then**
    $j \leftarrow 0 : \text{floor}(\frac{S-K}{N})$
    $P \leftarrow \text{sum}\left(\frac{1}{N^K}(-1)^j\binom{K}{j}\binom{S-Nj}{K}\right)$
    return P
  else
    $j \leftarrow 0 : \text{floor}(\frac{N*K-S}{N})$
    $P \leftarrow \text{sum}\left(\frac{1}{N^K}(-1)^j\binom{K}{j}\binom{K(N+1)-S-Nj-1}{K}\right)$
    return $1 - P$
  end if
end procedure

vectorized algorithm is extremely fast. It takes about half a second, for example, to calculate the exact p-value of all possible RS in the range $N \leq RS \leq N \cdot K$, for the problem $K = 5$ and $N = 10000$, typically encountered in microarray studies. Although the expression for $P(S \leq s;N,K)$ is algebraically correct, straightforward computation produces numerical errors whenever the dataset is too large. For such cases, the binomial coefficient that has s in the upper index becomes extremely large and storing large numbers for subsequent calculation creates numerical overflow due to the precision limitation of fixed-precision arithmetic, resulting in erroneous results (Dobrushkin, 2009, p: 369). There are remedies to address this problem. One is to use recurrence relations that

satisfy the generating function, discussed by various authors (Tsao, 1956; Bollinger, 1986, 1993; Dobrushkin, 2009, p: 370). However, the recursive algorithms we examined were computationally expensive to run, except for small to moderate values of N and/or K. An alternative and faster way of computing $P(S \le s; N, K)$ correctly is to use arbitrary-precision arithmetic computation to deal with numbers that can be of arbitrary large size, limited only by the available computer memory. Therefore, whenever the dataset is large the R code uses the package Rmpfr (Maechler, 2015) for high precision arithmetic to calculate the exact p-values. As one can see in **Table 1**, RankProd 2.0 computes the exact p-value for the RS in an extremely fast manner, for small to moderately large dataset sizes. For very large datasets, RankProd 2.0 switches to the Rmpfr version of the function drastically increasing the computation time (bold values). Unfortunately,

	N=10^2	10^3	10^4	10^5	10^6	10^7
K=5	0.006	0.0055	0.0053	0.0055	0.0054	0.0054
10	0.0056	0.0057	0.0056	0.0066	0.006	0.0059
20	0.0072	0.007	0.007	0.007	0.007	0.007
30	0.0097	0.0098	0.0094	0.0093	0.0093	0.0092
40	0.0117	0.0115	0.0114	0.0113	0.0113	0.0111
50	0.0136	0.0136	0.0134	0.0136	0.0133	**1.571**
60	0.0154	0.0154	0.0152	**1.686**	**1.7**	**1.705**
70	0.0175	0.0175	0.0174	**1.859**	**1.858**	**1.861**
80	**1.835**	**1.843**	**1.933**	**1.961**	**1.986**	**2.025**
90	**1.935**	**2.008**	**2.063**	**2.121**	**2.145**	**2.184**

Table 1. Computational time (in milliseconds) needed by RankProd 2.0 to compute the exact p-values for $RS = \text{trunc}(K * (N+1)/2)$. When the size of the dataset forces us to switch to the Rmpfr version of the function, the computation time drastically increases (bold values).

when dealing with extremely large datasets the time needed to compute the p-values quickly becomes impractical. As mentioned before, the RS distribution quickly tends to a Gaussian distribution. It is easy to show that with the Gaussian approximation, the largest relative errors are found in the tails, corresponding to the smallest p-values. Incidentally, the computational time needed to compute the p-values with the exact method is small when considering the smallest p-values (i.e. the smallest RS values). Knowing this, when the size of the dataset is such that the time needed to evaluate all the exact p-values becomes unacceptable, the new package uses the exact distribution only for the tails, whereas all the other p-values are evaluated through the Gaussian approximation. The switch between the two strategies happens after a user-defined amount of time.

2.1 Comparison with old approach

To assess numerical accuracy of the new implementation of the p-value evaluation we compared it with the permutation-based approach implemented in the old version of the package (**Fig 1**). In the permutation-based procedure the smallest possible p-value is equal to the inverse of the number of permutations. When getting close to this limit, the permutation approach becomes very imprecise (insets). Obtaining an accurate estimate for the complete range of all possible Rank Products (or Rank Sums)with the old implementation would require a prohibitive number of permutations.

3 Application to unpaired datasets

The Rank Products method was initially introduced for the analysis of gene expression in paired datasets, specifically two-color microarrays (Breitling *et al.*, 2004). In the old version of the RankProd package, an *ad*

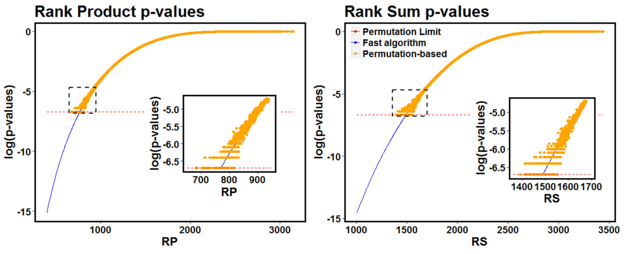

Fig. 1. Comparison of the p-values evaluation with the old permutation approach. Both for the RP and the RS the permutation-based approach becomes very imprecise when getting close to its limit (p = 1/(number of permutations*number of variables). Using the fast algorithm for the RP (and the exact calculations for the RS) provides precise p-values for the whole range of RP (or RS) values. For the illustration, 1000 permutations were used, but the general pattern is independent of the number of permutations used in realistic analyses.

hoc implementation able to cope with unpaired datasets is already present. Nevertheless, the number of unpaired datasets in molecular profiling experiments is constantly rising. This led to the need of a new and more principled approach. Let A and B stand for two experimental conditions in a molecular profiling experiment. In the dataset under study, the level of N features is provided for M_A samples in class A and for M_B samples in class B. When dealing with a paired experiment, the procedure to evaluate the fold changes needed to rank our metabolites is relatively straightforward. It can be easily noticed that, in the case of an unpaired dataset the ranking is not as trivial as before: which fold changes should be considered? In the old package, this problem was tackled by generating all the possible pairings of samples. In this way, the RP method (or the RS) is applied to a new dataset containing N features and $M_A \times M_B$ "replicates". It appears that the use of this approach leads to an increase of the number of false positives detected when compared with the paired case. A novel procedure called **Random Pairing** (described in **Algorithm 2**) has been implemented in the new package to address this issue.

Algorithm 2 Random Pairing

procedure Random Pairing
  A ▷ $(M_A \times N)$ data matrix group A
  B ▷ $(M_B \times N)$ data matrix group B
  $M_A \leftarrow \text{nrows}(A)$ ▷ number of samples in group A
  $M_B \leftarrow \text{nrows}(B)$ ▷ number of samples in group B
  npairs $\leftarrow \min(M_A, M_B)$ ▷ defining the number of pairs
  Randompairs $\leftarrow 1000$ ▷ defining the number of random pairings
  RPmatrix $\leftarrow$ matrix$(0, \text{nrow} = N, \text{ncol} = \text{Randompairs})$
  for pair in 1:Randompairs **do**
    indA $\leftarrow$ sample$(1 : M_A)[1 : \text{npairs}]$
    indB $\leftarrow$ sample$(1 : M_B)[1 : \text{npairs}]$
    data $\leftarrow$ rbind$(A[\text{indA},], B[\text{indB},])$
    RPmatrix[, pair] $\leftarrow$ RankProduct(data) ▷ evaluating the RP for the paired case
  end for
  RPvec $\leftarrow$ apply(columns, median, RPmatrix) ▷ the RP value assigned to each variable
  ▷ is the median of all the RPs evaluated in the for cycle
  return RPvec
end procedure

Instead of considering all the possible pairs at once, this procedure generates a user-defined number of random pairings (by default, this number is fixed as the minimum odd integer greater or equal to $M \times M$, where M is the minimum value of M_A and M_B) and evaluates the RP (or RS) statistics for each of them. Each of these randomly paired datasets has M samples. For each variable, the final RP (or RS) value returned is the median of all the values found during the random pairing process. The p-value evaluation is then performed as in the case of a paired experiment.

3.1 Comparison with old approach

The novel Random Pairing procedure handles unpaired datasets in a more principled way than the old *ad hoc* implementation. The p-value calculations of RankProd 2.0 for unpaired datasets are more rigorous than before, yielding more reliable significance estimates. As shown in **Fig 2**, the p-values for the new and old method are very highly correlated, but in the tails of the distribution the values for the old method were slightly smaller than for the new random-pairing method (insets), leading to a larger number of false positives than warranted.

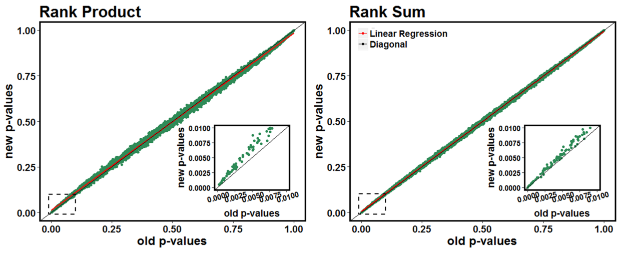

Fig. 2. Correlation between the p-values estimates between the two versions of the package. For both statistics the correlation is extremely high ($R^2_{adj} = 0.9989$ for RP and $R^2_{adj} = 0.9997$ for the RS). As can be seen in Fig 1, the permutation-based approach does not introduce a bias in the p-values, so these differences are not due to the way the p-values themselves are calculated.

References

Albert, J. (2002). Sums of uniformly distributed variables: a combinatorial approach. *College Mathematics Journal*, **33**(3), 201–206.

Balasubramanian, K., Viveros, R., and Balakrishnan, N. (1995). Some discrete distributions related to extended Pascal triangles. *Fibonacci Quarterly*, **33(5)**, 415–425.

Bellhouse, D. (2005). Decoding Cardano's *Liber de Ludo Aleae*. *Historia Mathematica*, **32**(2), 180–202.

Berry, D. A. and Lindgren, B. W. (1996). *Statistics: Theory and Methods. Second Edition*. Duxbury Press, Belmont, CA.

Bollinger, R. C. (1986). A note on Pascal-T triangles, multinomial coefficients, and Pascal pyramids. *Fibonacci Quaterly*, **24(2)**, 140–144.

Bollinger, R. C. (1993). Extended Pascal triangles. *Mathematics Magazine*, **66**(2), 87–94.

Breitling, R. and Herzyk, P. (2005a). Biological master games: using biologists' reasoning to guide algorithm development for integrated functional genomics. *Omics: A Journal of Integrative Biology*, **9**(3), 225–232.

Breitling, R. and Herzyk, P. (2005b). Rank-based methods as a non-parametric alternative of the t-statistic for the analysis of biological microarray data. *Journal of Bioinformatics and Computational Biology*, **3**(5), 1171–1189.

Breitling, R., Armengaud, P., Amtmann, A., and Herzyk, P. (2004). Rank products: a simple, yet powerful, new method to detect differentially regulated genes in replicated microarray experiments. *FEBS Letters*, **573**(1), 83–92.

Cardano, G. (1663). Liber de ludo aleae. In Cardus Sponius, editor, *Hieromymi Cardani Mediolanensis Opera Omnia*, pages 262–276. I.A. Huguetan, Lugdunum [Lyon].

DasGupta, A. (2010). *Fundamentals of Probability. A First Course*. Springer, New York.

de Moivre, A. (1711). De mensura sortis, seu, de probabilitate eventuum in ludis a casu fortuito pendentibus. *Philosophical Transactions (329)*, pages 213–264.

Dobrushkin, V. A. (2009). *Methods in Algorithmic Analysis*. Chapman and Hall/CRC, Boca Raton, FL.

Edgington, E. S. (1972). An additive method for combining probability values from independent experiments. *Journal of Psychology*, **80**(2), 351–363.

Eger, S. (2013). Restricted weighted integer compositions and extended binomial coefficients. *Journal of Integer Sequences*, **16**, 13.1.3.

Eisinga, R., Breitling, R., and Heskes, T. (2013). The exact probability distribution of the rank product statistics for replicated experiments. *FEBS Letters*, **587**(6), 677–682.

Ericksen, L. (2002). Sum of powers from uniform distribution moments. *Journal of Statistical Planning and Inference*, **101**(1-2), 57–79.

Feller, W. (1968). *An Introduction to Probability Theory and Its Applications. Volume I*. Wiley, New York.

Fielder, D. C. and Alford, C. O. (1991). Pascal's triangle: Top gun or just one of the gang? In H. A. Bergum G.E., Philippou A.N., editor, *Applications of Fibonacci Numbers Volume 4*, pages 77–90. Kluwer, Dordrecht.

Hald, A. (2003). *A History of Probability and Statistics and their Applications Before 1750*. Wiley, New York.

Heskes, T., Eisinga, R., and Breitling, R. (2014). A fast algorithm for determining bounds and accurate approximate p-values of the rank product statistic for replicate experiments. *BMC Bioinformatics*, **15**(1), 367.

Huygens, C. (1657). De ratiociniis in ludo aleae. In F. Van Schooten, editor, *Exercitationum Mathematicarum, Liber V*, pages 520–534. J. Elsevirius, Lugdunum Batavorum [Leiden].

Jeffery, I. B., Higgins, D. G., and Culhane, A. C. (2006). Comparison and evaluation of methods for generating differentially expressed gene lists from microarray data. *BMC Bioinformatics*, **7**(1), 359.

Johnson, N. L. and Kotz, S. (1977). *Urn Models and Their Application. An Approach to Modern Discrete Probability Theory*. Wiley, New York.

Johnson, N. L., Kemp, A. W., and Kotz, S. (2005). *Univariate Discrete Distributions*. Wiley, New York.

Koziol, J. and Feng, A. (2004). A note on the genome scan meta-analysis statistic. *Annals of Human Genetics*, **68**(4), 376–380.

Koziol, J. A. (2010a). Comments on the rank product method for analyzing replicated experiments. *FEBS Letters*, **584**(5), 941–944.

Koziol, J. A. (2010b). The rank product method with two samples. *FEBS Letters*, **584**(21), 4481–4484.

Levinson, D. F., Levinson, M. D., Segurado, R., and Lewis, C. M. (2003). Genome scan meta-analysis of schizophrenia and bipolar disorder, part 1: methods and power analysis. *American Journal of Human Genetics*, **73**(1), 17–33.

Luo, R., Hu, Z., Kong, W., Wen, S., and Xu, C. (2010). A rank-sum testing method for multi-trait comprehensive ranking and its application. *Agricultural Sciences in China*, **9**(8), 1117–1126.

Maechler, M. (2015). Rmpfr: R mpfr-multiple precision floating-point reliable. *R package version 0.6-0, URL http://rmpfr. r-forge. r-project. org*.

McShane, J. M. and Ratliff, M. I. (2003). Dice distributions using combinatorics, recursion, and generating functions. *College Mathematics Journal*, **34**(5), 370.

Murty, V. (1981). On dice-sum frequencies. *Two-Year College Mathematics Journal*, **12**(3), 209–211.

Neff, J. D. (1982). Dice tossing and Pascal's triangle. *Two-Year College Mathematics Journal*, **13**(5), 311–314.

Özkan, F. (1987). Some combinatorial problems related to rank sums in a two-way classification. *Communications, Faculty of Science, University of Ankara Series A1: Mathematics and Statistics*, **36**(2), 83–99.

R Core Team (2015). *R: A Language and Environment for Statistical Computing*. R Foundation for Statistical Computing, Vienna, Austria.

Reiczigel, J. (1999). Analysis of experimental data with repeated measurements. *Biometrics*, **55**(4), 1059–1063.

Rémond de Montmort, P. (1713). *Essay d'analyse sur les jeux de hazard (Seconde édition revûe & augumentée de plusieurs lettres)*. J. Quillau, Paris.

Shen, Z. and Marston, C. M. (1995). A study of a dice problem. *Applied Mathematics and Computation*, **73**(2-3), 231–247.

Singh, A. K., Dalpatadu, R. J., and Lucas, A. F. (2011). The probability distribution of the sum of several dice: slot applications. *UNLV Gaming Research & Review Journal*, **15**(2), 109–118.

Strube, M. J. and Miller, R. H. (1986). Comparison of power rates for combined probability procedures: a simulation study. *Psychological Bulletin*, **99**(3), 407–415.

Stuart, A. (1954). Limit distributions for total rank values. *British Journal of Statistical Psychology*, **7**(1), 50–51.

Takács, L. (1967). On the method of inclusion and exclusion. *Journal of the American Statistical Association*, **62**(317), 102–113.

Tsao, C. K. (1955). Rank sum tests of fit. *Annals of Mathematical Statistics*, **26**(1), 94–104.

Tsao, C. K. (1956). Distribution of the sum in random samples from a discrete population. *Annals of Mathematical Statistics*, **27**(3), 703–712.

Uspensky, J. V. (1937). *Introduction to Mathematical Probability*. McGraw-Hill, New York.

Wardlaw, W. P. (1991). The reason why dice rolls are symmetrically distributed. *American Mathematical Monthly*, **98**(7), 650.

Weisstein, E. W. (2016). Dice. `http://mathworld.wolfram.com/Dice.html`.

Whitfield, J. (1953). The distribution of total rank value for one particular object in m rankings of n objects. *British Journal of Statistical Psychology*, **6**(1), 35–40.

Wise, L., Lanchbury, J., and Lewis, C. (1999). Meta-analysis of genome searches. *Annals of Human Genetics*, **63**(3), 263–272.

Wise, L. H. and Lewis, C. M. (1999). A method for meta-analysis of genome searches: Application to simulated data. *Genetic Epidemiology*, **17**(S1), S767–S771.
